# The Human Tissue-Engineered Cornea (hTEC): Recent Progress

**DOI:** 10.3390/ijms22031291

**Published:** 2021-01-28

**Authors:** Louis-Philippe Guérin, Gaëtan Le-Bel, Pascale Desjardins, Camille Couture, Elodie Gillard, Élodie Boisselier, Richard Bazin, Lucie Germain, Sylvain L. Guérin

**Affiliations:** 1 CUO-Recherche, Médecine Régénératrice—Centre de Recherche du CHU de Québec, Université Laval, Québec, QC G1S 4L8, Canada; Louis-Philippe.Guerin@USherbrooke.ca (L.-P.G.); gaetan.lebel17@gmail.com (G.L.-B.); Pascale.desjardins.1@ulaval.ca (P.D.); Camille.couture.1@ulaval.ca (C.C.); elodie.gillard.18@gmail.com (E.G.); Elodie.boisselier@fmed.ulaval.ca (É.B.); bazin988@hotmail.com (R.B.); Lucie.germain@fmed.ulaval.ca (L.G.); 2 Centre de Recherche en Organogénèse Expérimentale de l’Université Laval/LOEX, Québec, QC G1J 1Z4, Canada; 3 Département d’Ophtalmologie, Faculté de Médecine, Université Laval, Québec, QC G1V 0A6, Canada; 4 Département de Chirurgie, Faculté de Médecine, Université Laval, Québec, QC G1V 0A6, Canada

**Keywords:** human cornea, tissue-engineering, 3D corneal model, wound healing, limbal stem cells, epithelium, stroma, endothelium

## Abstract

Each day, about 2000 U.S. workers have a job-related eye injury requiring medical treatment. Corneal diseases are the fifth cause of blindness worldwide. Most of these diseases can be cured using one form or another of corneal transplantation, which is the most successful transplantation in humans. In 2012, it was estimated that 12.7 million people were waiting for a corneal transplantation worldwide. Unfortunately, only 1 in 70 patients received a corneal graft that same year. In order to provide alternatives to the shortage of graftable corneas, considerable progress has been achieved in the development of living corneal substitutes produced by tissue engineering and designed to mimic their in vivo counterpart in terms of cell phenotype and tissue architecture. Most of these substitutes use synthetic biomaterials combined with immortalized cells, which makes them dissimilar from the native cornea. However, studies have emerged that describe the production of tridimensional (3D) tissue-engineered corneas using untransformed human corneal epithelial cells grown on a totally natural stroma synthesized by living corneal fibroblasts, that also show appropriate histology and expression of both extracellular matrix (ECM) components and integrins. This review highlights contributions from laboratories working on the production of human tissue-engineered corneas (hTECs) as future substitutes for grafting purposes. It overviews alternative models to the grafting of cadaveric corneas where cell organization is provided by the substrate, and then focuses on their 3D counterparts that are closer to the native human corneal architecture because of their tissue development and cell arrangement properties. These completely biological hTECs are therefore very promising as models that may help understand many aspects of the molecular and cellular mechanistic response of the cornea toward different types of diseases or wounds, as well as assist in the development of novel drugs that might be promising for therapeutic purposes.

## 1. Introduction

Vision is crucial when it comes to our interactions with our surroundings. In order to see, light must be refracted by the cornea, the outer layer of the eye, onto the lens, and then reach the retina. Thus, our visual abilities rely heavily on corneal transparency and its refractive power. The cornea accounts for two-thirds of the overall refractive power of the eye and provides defense against trauma and infections. It is composed of three layers: the epithelium, the stroma, and the endothelium, which are divided by two extracellular matrix interfaces: the Bowman’s layer and the Descemet membrane [1]. The epithelium can readily regenerate and restore normal vision after a minor trauma. However, in the case of deeper wounds, the stroma tends to develop permanent opaque scars, the extent of which is related to the severity of the injury. The endothelium cannot regenerate in humans under normal conditions. The principal physiological function of these cells is to allow the leakage of solutes and nutrients from the aqueous humor to the more superficial layers of the cornea, while at the same time pumping water in the opposite direction. When endothelial cells are damaged, the cornea swells, loses transparency, and develops bullae on its anterior surface.

### 1.1. Anatomy of the Human Cornea

**Figure 1:**
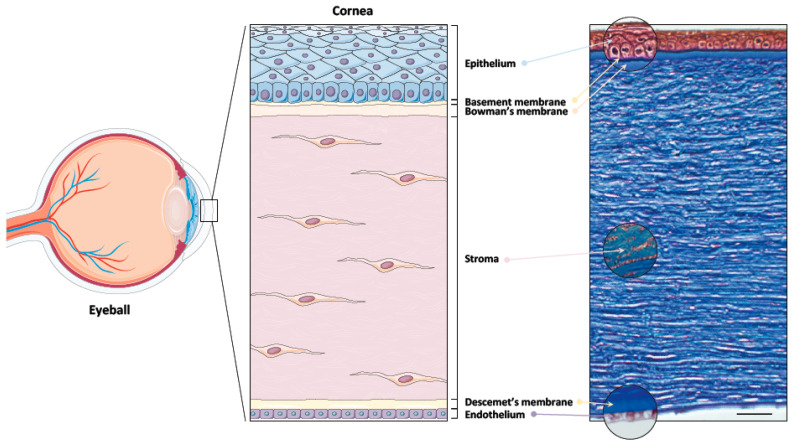
Schematic of the human cornea and histology. **Left** panel: Schematic view of the human eye. The cornea forms the transparent front part of the eyeball. **Central** panel: From anterior to posterior, the cornea is made up of a stratified squamous epithelium deposited on a basement membrane, follows the Bowman’s membrane, a stroma, composed predominantly of collagen fibrils in which keratocytes are entangled, the Descemet’s membrane, and a monolayer of endothelial cells. **Right** panel: Masson trichrome staining of a section of the entire native human cornea showing all cellular compartments of that tissue. Scale bar: 50 μm.

The human cornea is a transparent avascular tissue and is the most anterior structure of the eye. Its three main purposes are to protect the intraocular contents, to allow light to enter the eye and reach the retina, and to provide two thirds of the refractive power of the optic system [2]. Many factors contribute to corneal transparency: the regularity of the surface and thickness of the epithelium in association with the integrity of the lacrimal film, the regular architecture of the collagen fibrils in the stroma, the production of soluble crystallin proteins and the presence of proteoglycans produced by the stromal keratocytes, the regulation of the hydration level by the endothelium, and the absence of vascularization [3,4,5,6,7]. The diameter of the cornea is approximately 12 mm and the mean anterior corneal curvature radius is around 8 mm [8]. The corneal thickness is about 540 µm in the center and 700 µm in the periphery. Being avascular, the cornea’s nutritional supply (mostly oxygen and glucose) comes from small blood vessels in the corneoscleral junction as well as from palpebral arteries through the aqueous humor and the lacrimal film [1,9]. Moreover, it is estimated that the cornea has an innervation density 300–600 times that of the skin and 20–40 times that of the tooth pulp, making it one of the most innerved tissues in the human body [10,11]. The five components of the cornea (from anterior to posterior: corneal epithelium, Bowman’s membrane, corneal stroma, Descemet’s membrane, and corneal endothelium) are described below [2] (also refer to Figure 1).

#### 1.1.1. Corneal Epithelium

The epithelium is the outermost layer of the cornea and is thus constantly exposed to the environment and the lacrimal film. The epithelium is around 40–50 µm thick and it is the most important refractive element of the eye. It is composed of 5–7 layers of stratified and nonkeratinized squamous epithelial cells resting on a basement membrane produced by both basal epithelial cells and keratocytes from the stroma [1]. The basement membrane is 40–60 nm thick and is composed of type IV collagen, laminin, perlacan, and nidogen [12]. The epithelium can be further separated into 3 cell layers: the superficial cells, the wing cells, and the basal cells. The superficial epithelial cells form 2–3 flat layers and act as a barrier that prevents toxins, microorganisms, and tears from entering the epithelium. This is the result of the tight junctions between these cells. Moreover, on top of the outermost cells is the glycosylax, which is essential to the stability of the tear film. The epithelial cells are eliminated by desquamation and their turnover is assured by the basal layer. Basal cells divide and differentiate into wing and superficial epithelial cells as they migrate towards the surface [13]. They are connected to the basement membrane by hemidesmosomes while epithelial cells are linked to their neighbors by lateral gap junctions and zonulae adherens [14]. The lifespan of epithelial cells is 7–10 days. The epithelium completely renews itself every week through centripetal migration starting from the limbus, which is located at the corneoscleral junction and is where the corneal epithelial stem cells are found [15,16]. Stem cells divide asymmetrically into transient amplifying cells, which migrate to form the basal cell layer of the central cornea.

#### 1.1.2. Bowman’s Layer

Bowman’s membrane is an acellular layer between the basement membrane of the epithelial cells and the corneal stroma. It is primarily composed of collagen type I, III, V, VI, and XII, but also contains collagen type IV and VII coming from the basement membrane [17,18,19,20]. This collagen is organized in small fibrils of 20–25 µm in diameter. The fibrils interconnect to form an 8–12 µm thick layer of tissue. The thickness of the Bowman’s membrane has been shown to decrease with age. Moreover, this membrane does not regenerate after trauma or surgical removal, which leads to scarring [17,21]. The function of this structure is still unclear as its absence does not result in loss of vision or in important structural changes in the cornea. It is hypothesized that the Bowman’s membrane could participate in the protection of the sub-epithelial nervous plexus, which goes through the anterior stroma [17].

#### 1.1.3. Corneal Stroma

Accounting for roughly 90% of the cornea’s thickness, the stroma plays a pivotal role as it provides major structural support and crucial properties such as tensile strength, stability, and transparency [12]. It is composed mainly of fibrils of collagen types I and V organized in lamellar sheets, but collagen types III, VI, XII, and XIV can also be found [22,23]. Additionally, these sheets are surrounded by proteoglycans associated with glycosaminoglycans that are decorated with chondroitin sulfate or keratan sulfate, and this assures the hydration and transparency of the cornea [24]. The stroma contains 250–300 collagen lamellae that are heterogeneously organized. In the anterior stroma, the collagen fibers are interwoven, whereas in the mid to posterior stroma, they are parallel to each other [25]. Keratocytes, which are responsible for the renewal of the stroma, are located between the lamellae and occupy around 20% of the stroma. Their role is to produce collagen and proteoglycans, thus contributing to the stability of the stromal framework as well as to the regulation of the extracellular matrix and to the wound healing process [26,27,28]. The transparency and biochemical properties of the cornea are the result of the stroma’s complex architecture.

#### 1.1.4. Descemet’s Membrane

The Descemet’s membrane is a basement layer that is continuously secreted by corneal endothelial cells and which maintains the endothelium’s structure. Its thickness is around 3 μm at birth but, contrary to the Bowman’s membrane, expands to approximately 10 μm with age. Produced before birth, the anterior portion of the Descemet’s membrane (in contact with the stroma) is fibrous. In contrast, the posterior portion (in contact with the endothelium) is produced after birth, and is more homogenous [11,29,30]. Structurally, the Descemet’s membrane mainly contains fibrils of collagen types IV and VIII, although collagen types III, V, and VII can also be found, as well as fibronectin and laminins [19,20,31,32]. As the basement membrane of the endothelium, the Descemet’s membrane participates in corneal dehydration. This is evident in corneal hydrops, which is a possible complication in advanced keratoconus and is characterized by stromal oedema following the tearing of the Descemet’s membrane [33,34].

#### 1.1.5. Corneal Endothelium

The human corneal endothelium is organized in a single layer of hexagonal cells covering the posterior face of the cornea. This 5 μm thick layer forms a relatively uniform and regular mosaic and is in direct contact with the aqueous humor. The endothelial cells have a diameter of 20 μm and are responsible for maintaining the deturgescent state of the corneal stroma via ionic pumps situated in the basolateral portion of their cellular membranes [11]. Endothelial cells possess lateral gaps and tight junctions and are attached to the Descemet’s membrane by hemidesmosomes. This results in the formation of a barrier that allows the passage of water from the aqueous humor to the stroma [35,36,37]. Contrary to the corneal epithelial cells, endothelial cells are incapable of mitosis and therefore cannot regenerate in vivo. The replacement of damaged cells is provided by the centripetal migration of peripheral endothelial cells [38,39,40]. The cell density, initially around 4000 cell mm^−2^, decreases with age at a rate of approximately 0.6% per year in the central endothelium to reach a density of 2500 cell mm^−2^ in adults. Moreover, this phenomenon is accompanied by an increase in the surface area covered by each cell [41,42,43]. The main function of the endothelium is to control stromal dehydration in order to prevent the formation of corneal oedema, which leads to the loss of transparency and thus vision. This is the result of Na+, K+–adenosine triphosphatase (ATPase) pumps located in the basolateral membrane. These pumps passively move ions and water from the stroma, which is hypotonic, to the aqueous humor, which is hypertonic [44,45]. Consequently, this mechanism is crucial for maintaining transparency and for nourishing the cornea, as the ion and water exchanges allow the transfer of nutrients.

### 1.2. Clinical Aspects of Corneal Blindness and Current Therapeutic Strategies

Due to its anatomical location, the cornea is continuously exposed to abrasive forces and mechanical, chemical, and thermal trauma. Corneal diseases represent the fifth leading cause of blindness around the globe, preceded by cataracts, refractive errors, glaucoma, and age-related macular degeneration [46]. According to the first global report on vision launched in November 2019 by the World Health Organization (WHO), at least 2.2 billion people worldwide suffer from a vision impairment or blindness (https://www.who.int/publications-detail/world-report-on-vision). The vision impairment of more than one billion of these patients, of which 4.2 million impairments are due to unaddressed corneal opacities, could have been prevented or have yet to be addressed. In 2012, it was estimated that 12.7 million people were waiting for a corneal transplantation worldwide. Unfortunately, only 185,000 cornea transplantations could be performed in the world the same year, covering the needs of a mere 1 in 70 patients [47]. Furthermore, according to Matthew et al., it is estimated that 4.9 million persons worldwide suffer from bilateral corneal blindness and could potentially benefit from a corneal transplant [48]. Of these individuals, a large proportion (near 90%) live in underdeveloped countries and are significantly younger than the individuals suffering from other causes of blindness [48]. The etiologies of corneal blindness vary greatly from country to country. Accordingly, the indications for corneal transplantation are mainly based on geographical, political, and economic factors [49]. In 2016, the 4 main indications for corneal transplantation were Fuch’s dystrophy, pseudophakic bullous keratopathy, repeat corneal transplantation, and keratoconus [49].

Ocular traumas and corneal ulcerations could be the cause of up to 1.5–2 million new cases of monocular blindness every year [50]. Diseases such as trachoma, accounting for 4.9 million cases of blindness (WHO), Fuch’s dystrophy, xerophtalmia, onchocerciasis, and ophthalmia neonatorum are known to lead to corneal blindness via scarring and vascularization [51]. Scarring can also occur following chemical burns, especially alkaline burns. Complicated ophthalmic surgeries as well as chronic inflammation may also lead to long-term corneal endothelial decompensation.

In developed countries, easy access to fresh corneal tissue combined with new surgical techniques (deep anterior lamellar keratoplasty (DALK), Descemet’s stripping endothelial keratoplasty (DSEK), Descemet’s stripping automated endothelial keratoplasty (DSAEK), Descemet membrane endothelial keratoplasty (DMEK), conjunctival-limbal autografting (CLAu), simple limbal epithelial transplantation (SLET), cultivated limbal epithelial transplantation (CLET), etc.) has made visual rehabilitation following corneal transplantation very successful. The traditional penetrating keratoplasty (PK) represented only 34% of corneal transplantations in the United States in 2018, whereas endothelial keratoplasties accounted for 59%, and 7% were anterior lamellar keratoplasties (ALK) or other types of graft (Eye Bank Association of America, 2018 Statistical Report; https://restoresight.org/what-we-do/publications/statistical-report/). In PK, the overall observed graft survival rate has been similar in many studies over the past 15 years and revolves around 87–96%, 63–76%, and 52–64% at 1, 5, and 10 years, respectively [52,53,54]. The advent of post-PK refractive surgery and the development of new types of contact lenses (hybrid and scleral contact lenses) have also greatly improved the visual outcome following this procedure. For endothelial keratoplasties, the graft survival rate is 93% at 5 years [55]. For most of these procedures, post-operative vision can be corrected with simple glasses in a matter of a few months since they do not create large amounts of astigmatism. Despite the excellent results obtained for a first corneal transplantation in patients not at high risk, the graft survival rates drop significantly in high-risk patients, including those afflicted with chemical burns, autoimmune diseases, or with a history of previous graft rejections. For these patients, the rejection rate may rise as high as 49%, which might lead to repeat surgeries and worsening of the prognosis after each graft [52,56,57].

The actual cornea shortage worldwide strikes mainly in underdeveloped countries, where, as aforementioned, 90% of corneal blindness is observed. It is estimated that 3.5 million people are in need of a corneal transplant, whereas only 20,000 donors are reported every year [58]. Therefore, it is clear that alternatives to cadaveric corneas must be turned to in the future.

One of such alternatives is the keratoprosthesis, which is a device that uses clear polymers (acrylic, polymethyl methacrylate (PMMA), hydroxyethyl methacrylate (HEMA), etc.) embedded in the cornea to reestablish the transparency of the central cornea. The difficulties in creating keratoprosthesis come from the need for biocompatibility with the plastic material used, the long-term integration of such polymers within living tissues, and the permanent exposure to the air of the outer component of the keratoprosthesis. Throughout the years, different strategies have been developed to address these difficulties: the creation of an intrastromal pocket to receive the implant (AlphaCor (Coopervision Surgical Inc., Lake Forest, CA, USA), KeraKlear (Keramed, Inc., Sunnyvale, CA, USA), etc.), the insertion of the device in bone tissue surrounded by a fibrovascular membrane (osteo-odonto keratoprosthesis (OOKP), modified OOKP (MOOKP)), and the placement of the clear prosthetic cylinder in the center of a corneal graft with front and back plates that snap together with corneal tissue sandwiched in-between (Boston K-Pro) [59,60,61,62,63,64]. Keratoprosthesis are usually last-resort procedures used when conventional corneal transplantation does not reestablish vision satisfactorily.

The collagen-based hydrogel is another option for the actual shortage of cadaveric cornea [65,66,67]. The extracellular matrix (ECM) of the corneal stroma is primarily composed of collagen type I with some collagen types III [22], V, VI, XII, XIII, XIV, and XXIV [67]. Recombinant human collagen types I and III (collagen type III having a smaller fibril diameter and thus possibly making a more compact and robust hydrogel [68]) were studied and compared as mimics to the collagen-based corneal stroma. Both types were proven to possess similar physical and chemical properties to corneal substitutes, but the synthetic cross-linked recombinant human collagen type III (RHC-III) was found to be superior regarding optical clarity [67]. Over the years, it was modified by the addition of 2-methacryloyloxyethyl phosphorylcholine (MPC) to further enhance its stability, mostly the enzymatic resistance, and to prevent neovascularization while still allowing regeneration [69], making it an interpenetrating collagen-phospholipid network (RHC-III-MPC) [70]. Recombinant human collagen corneal implants may offer an interesting alternative for patients in need of an anterior lamellar keratoplasty. However, their lack of mechanical strength precludes their use for keratoplasties requiring a thicker stromal graft.

Fibrin, a non-globular fibrous protein that participates in blood clotting, has also been studied for corneal transplantation. In 1997, Pellegrini et al. confirmed that it could be used for the production of autologous, limbal stem cell-derived corneal epithelial sheets that can then be used for transplantation in patients with corneal epithelial defects [71]. Since then it has been thoroughly tested, showing that the limbal stem cells are maintained when cultivated on a fibrin substrate and that in more than 75% of cases at 10 years post-transplant [72,73], the corneal integrity of patients suffering from total limbal stem cell deficiency (LSCD) unresponsive to surgical therapy can be restored permanently when the transplantation of limbal stem cells cultured on a fibrin gel is performed. Fibrin also has many other applications, the most noteworthy in ophthalmology being fibrin glue for wound closure in numerous surgical procedures, where it often replaces sutures for an enhanced cosmetic result [74,75]. However, it has been shown that fibrin glue delays corneal epithelial cell growth and migration and should therefore be used with caution [76]. Recently, Dereli Can et al. published a study in which they showed that their human-derived platelet-rich fibrin (HPRF) released significantly more growth factors than the traditional human amniotic membranes (HAMs) when used as a substrate for limbal explant culture [77]. Moreover, it proved better than the HAMs for cell proliferation, migration, and stemness, as well as being an inexpensive biomaterial for the optimization of limbal explant culture [77].

Fibrin-based corneal implants have proven useful for further improving the adhesion, migration, and stemness of limbal stem and progenitor cells [77] as well as their long-term survival during the cultivation process [78].

Frequently used in medicine nowadays, human amniotic membranes (HAMs) have several physical and chemical properties that are applicable to modern ophthalmic issues. Among them are the stimulation of epithelialization and differentiation of epithelial cells as well as the enhancement of adhesion and the prevention of apoptosis [79,80,81]. Moreover, HAMs possess anti-fibrotic and anti-inflammatory properties, mainly via the suppression of TGF-beta signaling and the inhibition of pro-inflammatory cytokines, respectively [82]. They also produce several anti-angiogenic compounds [83] and anti-microbial agents [84]. Finally, HAMs’ immunomodulatory effect (with close to no risk of rejection due to the low amount of class Ia and Ib human leucocyte antigens (HLA) present, therefore requiring no immunosuppression in HAM grafts [85,86]) and their transparency are two of their best assets. Indications for a HAM graft include corneal surface disorders with or without partial LSCD, such as persistent epithelial defects (PEDs) or non-healing corneal ulcers, conjunctival surface reconstruction after the removal of large lesions (malignant tumors, pterygium, symblepharon, etc.), glaucoma (as an adjunct therapy to reduce scarring at the time of filtering surgery and complication rates), scleral melts, and perforations. For non-traumatic micro-perforations and deep corneal ulcers with descemetoceles, 81.8% of grafts were successful at a mean follow-up time of 32 months (12 to 60), although 15% of the treated eyes developed complications [87]. It was shown to be useful in alleviating pain in 94% of patients with bullous keratopathy, [88], and in 93% of patients with band keratopathy [89]. HAMs have proven to be valuable adjuncts in some procedures and can be used alone to treat several ocular pathologies such as partial LSCD [90], PEDs, and corneal ulceration, to name a few.

When corneal epithelial stem cell autografts are impossible due to a bilateral condition such as LSCD, allografts may be used. However, the risk of pathogen transmission and rejection is much higher [91]. Another option is to find another stem cell population from the patient’s own cells as an alternative to the use of allogenic material. In the past years, many possible sites have been explored such as oral mucosa epithelial cells (OMECs), conjunctival epithelial cells, dental pulp stem cells, and hair follicle bulge stem cells. OMECs have proven to be a good replacement for corneal epithelial cells in ocular surface reconstruction as they share some physical properties and functions [92,93]. The cells are typically cultured on HAMs until stratification of the epithelium is achieved, then grafted. OMECs have demonstrated their ability to regenerate an avascular, stable, and epithelialized corneal surface in patients with severe LSCD [94,95]. Conjunctival epithelial cells grown on HAMs have also been used clinically in LSCD patients, but the results are poorer than those obtained with autologous limbal stem cells [96].

## 2. Tridimensional (3D) Scaffold Models of the Cornea

In the intent to develop new corneal replacements, two-dimensional (2D) models, where cell organization is provided by the substrate, are being replaced by their tridimensional (3D) counterparts. By improving tissue development and cell arrangement, these 3D scaffolds prove to be a closer imitation of the native human corneal architecture. Many materials have been studied with the goal of replicating the human cornea: silk, chitin, collagen-chitosan hydrogels, fibrin-agarose hydrogels, and polyacrylic acid hydrogels are some of the most promising models.

### 2.1. Silk-Based Corneal Implants

Silk, produced by silkworms and spiders, is a biomaterial well-known for its biocompatibility and low immunogenicity [97], its degradability [98], its optical transparency [99], and its tensile strength, which is superior to any other known synthetic fiber [100]. For these reasons, it has been used for medical purposes for a long time, more specifically for surgical sutures [101]. Moreover, silk provides a substrate that promotes substantial cell attachment and proliferation [102,103,104] and silk films with different surface groove patterns not only allow cell attachment and growth but also direct alignment of their ECM components, such as collagen type V and proteoglycan (decorin and biglycan) [103,104,105]. Silk fibroin is a structural fibrous protein extracted mainly from the cocoons of the silkworm *Bombyx mori*. Its physical properties have proven to be comparable to those of the human corneal stroma [106]. Many researchers have therefore tried using silk in vitro and in vivo in rabbit corneas as a substitute to allogenic corneal materials [107,108].

In 2010, Gil et al. functionalized silk biomaterials with arginylglycylaspartic acid (RGD) peptides, resulting in an improvement in cell attachment, proliferation, and alignment, as well as increased expression of corneal stroma markers. Their work also showed that their model could sustain the growth of corneal epithelial and stromal cells [109]. A year later, Higa et al. demonstrated that porous silk membranes were promising scaffolds for cultured epithelial sheets, providing regeneration of the corneal epithelium in rabbit models [110]. More recently, Wang et al. developed a porous silk-based corneal model that included the corneal epithelium and stroma with an artificial innervation mimicked by chicken dorsal root ganglion neurons [111]. Human corneal stromal stem cells and epithelial cells were seeded on the silk scaffolds and cultured either immersed in liquid medium or raised to the air–liquid interface in order to compare both conditions. After 28 days of cultivation, transparency was similar to that of a porcine cornea, with a higher axon density when cultivated at the air–liquid interface. Moreover, the immersed condition showed a decrease in the number of corneal stromal stem cells in comparison with culture at the air–liquid interface after adding the epithelial cells. The authors concluded that culture at the air–liquid interface should be used to create such a corneal model [111].

Silk has also been studied as a carrier for corneal endothelial cells. Both human and rabbit cells were cultured successfully on silk films and rabbit endothelial cells were even grafted during a Descemet membrane endothelial keratoplasty procedure conducted on New Zealand white rabbits, with promising results [112]. Moreover, it is worth mentioning that progress has been made in retinal reconstruction with silk scaffolds for the culture of retinal pigment epithelial cells [113], as well as in ocular wound healing and drug delivery (as reviewed in Reference [114]. Therefore, silk fibroin shows great promise for tissue engineering of the cornea. Often overlooked because of its supposed allergenic properties, sericin, another silkworm protein, has recently been studied as a substrate for cell growth. It has been found that silk sericin has a stronger cell attachment capacity when compared to fibroin, although its mechanical attributes were inferior [115].

### 2.2. Chitin-Based Corneal Implants

Chitin, a long-chained polymer derived from glucose, can be found naturally in arthropod exoskeletons and fungal cell walls. It can be converted into chitosan upon deacetylation [116]. Because they are biodegradable and biocompatible, chitosan and its derivatives have been widely used in tissue engineering as drug delivery systems [116,117]. In ophthalmology, in situ chitosan gels have been studied as a replacement for ocular drops essentially because they increase bioavailability and retention time as well as the compliance of patients by reducing the frequency of administration [118]. Chitosan has also been studied as a replacement for the amniotic membrane as a scaffold for corneal surface and endothelial reconstruction. It was found to be comparable to HAM regarding the preservation of the phenotype of bovine corneal epithelial cells but also proved to be superior concerning corneal epithelial cell proliferation and attachment rapidity [119].

According to Liang et al., reconstruction of the corneal endothelium could be possible using an in situ-formed hydrogel of hydroxypropyl chitosan (HPCTS) cross-linked with sodium alginate dialdehyde (SAD). By encapsulating corneal endothelial cells within such a HPCTS/SAD hydrogel that was then transplanted on the exposed Descemet’s membranes of rabbits’ corneas, they demonstrated its non-toxicity, histocompatibility, and quick biodegradability. Moreover, their experiment showed that the grafted endothelial cells could survive and retain their native morphology [120]. In 2014, Liang et al. studied a water-soluble derivative of chitosan, hydroxyethyl chitosan (HECTS), as a scaffold for the repair of mechanical damage in rabbit corneas. Its use resulted in an acceleration of re-epithelialization, as the repair time was cut in half when compared with the control group [121]. More recently, a hydrogel based on carboxymethyl chitosan (CMCTS) coupled with SAD was used to treat severe alkali burns of the ocular surface. Indeed, grafting limbal stem cells encapsulated with CMCTS/SAD hydrogel on the injured corneal surface led to quick wound healing and reconstruction of the burned tissues [122]. Overall, chitosan has shown great potential in tissue engineering, although it serves best when cross-linked with other molecules to enhance its basic properties.

### 2.3. Collagen-Chitosan Hydrogels

Chitosan, as discussed above, has many physical attributes that could benefit the field of tissue engineering in ophthalmology. Among them are its biocompatibility, its non-toxicity, and its biodegradability. This polymer also has notable cell adhesion properties as well as permeability and tensile strength [123]. However, its hydrophilic nature and reasonably low stability considerably restrict its use [124]. Therefore, many have tried combining chitosan with other materials such as nano-whiskers [124], poly(vinyl alcohol) [125], or collagen [123] in order to eliminate its weaknesses. In 2014, Li et al. developed a biomacromolecule containing chitosan and collagen cross-linked with 1-ethyl-3-(3-dimethylaminopropyl) carbodiimide (EDC). Upon analysis of this membrane, they concluded that the addition of collagen to a chitosan-based membrane greatly enhanced its physical attributes such as hydrophilicity and transparency. Furthermore, besides its ability to support cell adhesion, this model also proved to be biocompatible. Meanwhile, no adverse effects were noted in the culture of human corneal epithelial cells [126]. In vivo studies in pig and rat models have shown that collagen-chitosan hydrogels provide transparency and biological performance equivalent to HAMs as well as the promotion of re-epithelialization. Moreover, the results suggest that these scaffolds are implantable and bioresorbable and possess significant physical and biological properties that could potentially lead to large-scale production of these inexpensive nanofibrous materials [127,128]. However, although collagen-chitosan hydrogels sound promising for tissue engineering, human clinical trials as yet remain to be completed.

### 2.4. Fibrin-Agarose Hydrogels

As described in Section 1.2, fibrin is a protein involved in blood clotting and has been utilized for many purposes in tissue engineering during the last decades. In comparison with collagen type I, commonly used as a corneal stroma substrate, fibrin is inexpensive, readily available, and stable. Moreover, fibrin is not as quickly degraded and has a good affinity for corneal stromal cells [129,130,131]. Agarose hydrogels have not been extensively studied due to the slow cell growth rate they provide [132]. However, a fibrin-agarose scaffold yields a better consistency than fibrin alone, is much easier to manipulate (enough to be sutured), and provides good transparency as well. Additionally, these constructs do not shrink as much as collagen gels and are able to sustain a layer of rabbit corneal epithelial cells [133,134]. In 2011, Cardona et al. compared the transparency of fibrin and fibrin-agarose scaffolds at different time intervals up to 28 days of culture. Their study showed that the transparency, scattering, and absorption properties of both constructs were similar to those of the native human cornea. No superiority of the fibrin-agarose hydrogel was demonstrated. Nonetheless, the authors also mentioned that it could reduce the corneal keratocyte growth rate in vitro [135]. A clinical trial testing artificial human corneas bioengineered using fibrin-agarose nanostructured sheets and human allogeneic corneal stromal and epithelial cells, with the intention to treat severe corneal trophic ulcers, was recently initiated. Currently in phase I/II, preliminary results show good feasibility and safety [136].

### 2.5. Polyethylene Glycol and Polyacrylic Acid Hydrogels

Interpenetrating polymer network (IPN) hydrogels based on polyethylene glycol (PEG) and polyacrylic acid (PAA) were developed in 2007 by Myung et al. and used as corneal substitutes. These hydrogels proved to be biocompatible and had a tensile strength adjustable from roughly 10 kPa to 10 MPa. Moreover, their near 90% water content allows the passage of important nutrients such as glucose at a diffusion rate similar to that of the native cornea [137,138,139,140]. Inspiring results were also obtained from PEG corneal inlays successfully implanted into rabbit corneas [141,142]. Two years later, Parke-Houben et al. developed a biocompatible PEG/PAA interpenetrating polymer network (IPN) hydrogel with a porous periphery to allow better integration of the synthetic material into the native cornea. This model also supported extensive corneal fibroblast adhesion and proliferation [140]. However, the first study that assessed the biocompatibility of the PEG/PAA hydrogels on rabbits at 6 months also reported that a majority of them had complications ranging from a haze and epithelial defects to implant extrusion [143]. Therefore, PEG/PAA IPN hydrogels display interesting properties such as nutrient permeability as well as strength and transparency similar to those of the native cornea, but future clinical trials will be necessary to assess it as tissue-engineered corneal substitutes for long-term corneal replacement in vivo.

## 3. The Human Tissue-Engineered Cornea (hTEC)

As stated in the above section, the progress made in the field of tissue engineering has greatly increased and diversified the corneal substitutes available today for in vitro studies and in development for clinical purposes. However, many of these models rely on the use of exogenous material, which may raise concerns at the time of grafting due to their allogenic nature or pro-inflammatory properties. Therefore, cell-based and scaffold-free models have emerged.

Among them, the work of Zieske and Karamichos is worth mentioning. By culturing both rabbit corneal epithelial cells and immortalized mouse corneal endothelial cells on each side of a collagen matrix made of rabbit stromal fibroblasts, Zieske’s group first demonstrated that the production of a three-dimensional corneal construct with the three cell types found in the native cornea could be achieved [144]. Since then, other models based on the principle of self-assembly (the same principle used for our model, see Section 4.2) have been developed in order to study physiological processes in vitro. For instance, a self-assembled 3D model of the corneal stroma was developed using primary human corneal fibroblasts [145,146]. This model proved to be a useful tool for studying diabetes and keratoconous disease, among other things [147,148]. Originally much thinner than the native corneal stroma, this 3D stromal construct was recently improved in order to better mimic the corneal stroma in terms of size [149]. This corneal stromal construct was also upgraded with the addition of human corneal endothelial cells and primary and immortalized human corneal epithelial cells in two separate studies conducted last year [150,151]. All of this work points out the importance and potential of such multi-cellular corneal 3D models for in vitro studies.

### 3.1. The 3D Human Tissue-Engineered Cornea Generated by the Self-Assembly Method

Besides the need for a suitable model for research studies, the development of a model that could serve as an alternative to donor corneas for grafting purpose would be of great interest. For over 20 years now, we have been dedicating our efforts to producing a human tissue-engineered cornea (hTEC) that would be made exclusively of human primary corneal cells and that would faithfully mimic the human native cornea. Bearing this in mind, and with the aim of improving existing models, we successfully achieved the production of a human tissue-engineered cornea (hTEC) that is compatible for grafting and made up exclusively of untransformed human primary-cultured corneal cells. The reconstruction procedure, characteristics, and uses of this model are detailed in the following sections.

#### 3.1.1. Isolation and Culture of Human Corneal Cells (Epithelial, Stromal, and Endothelial Cells)

Corneal epithelial, stromal, and endothelial cells can be isolated and expanded from human donor corneas that are unsuitable for transplantation. In our case, corneas are obtained from our local eye bank (Centre Universitaire d’Ophtalmologie Eye bank, Québec, QC, Canada). Briefly, the Descemet membrane with its attached corneal endothelial cells is peeled off from the whole cornea and then subjected to digestion with collagenase A (or ethylenediaminetetraacetic acid (EDTA)) [152]. Cells are grown until they reach confluence in a proliferation medium (Opti-MEM-I medium supplemented with 8% fetal bovine serum, 5 ng/mL epidermal growth factor, 0.08% chondroitin sulfate, 20 μg/mL ascorbic acid, and penicillin/streptomycin). The culture medium is then switched to a maturation medium (Opti-MEM-I medium supplemented with 8% fetal bovine serum and penicillin/streptomycin) when endothelial cells reach complete confluence, and cells are grown for an additional 7 to 28 days. This two-phase culture has proven to be efficient for maintaining and improving the corneal endothelial cell phenotype [153]. Once the Descemet membrane has been peeled off, the remaining corneal tissues (stromas with their attached epithelial cells) are then incubated with dispase. The epithelium is mechanically separated from the underlying stroma and treated with trypsin, and hCECs are seeded with either lethally irradiated murine Swiss-3T3 fibroblasts (i3T3) or human dermal fibroblasts (iHFL) that are used as feeder layers (see Section 3.1.4 for recent improvements brought to the hTEC model). Human corneal fibroblasts (hCFs) are isolated from the stromal portion of the cornea that is left after dispase digestion and removal of the epithelium by incubating with collagenase H. All three cell types are passaged or cryopreserved in 90% *v/v* fetal calf serum and 10% *v/v* dimethyl sulfoxide (DMSO), aliquoted into cryogenic vials, and frozen at −80 °C overnight. Cells are then transferred and stored in liquid nitrogen until use.

#### 3.1.2. The Self-Assembly Procedure for the Reconstruction of hTECs

The first evidence that corneal fibroblasts could produce collagen in culture when under appropriate growing conditions goes back to the late 1970s [154]. Such a discovery suggested that it might be possible to produce, upon fibroblast stimulation, a 3D scaffold-free construct made of natural corneal ECM components. Along with the improvements in fibroblast isolation and culture procedures, this hypothesis was further validated in 1998 with the development of the first self-assembly approach by the Laboratoire d’Organogénèse expérimentale (LOEX) for blood vessels and skin [155,156]. The self-assembly approach was first developed as an alternative to the use of exogenous materials for tissue engineering. Since its development in 1998, this procedure has been refined and adapted, and now allows the reconstruction of various human tissues and organs (see below) that, besides being devoid of any added biomaterials, can be entirely autologous (when used as a bilamellar tissue constituted of both the corneal epithelium and stroma) and therefore compatible with grafting without rejection. The self-assembly technique relies on the natural ability of certain types of cells to organize a tri-dimensional tissue, in all respects similar to their native environment, when cultured under appropriate conditions. The presence of ascorbic acid in the culture medium efficiently induces spontaneous secretion and assembly of the ECM by fibroblasts [157,158]. Ascorbic acid promotes ECM production in two different ways: first, it is known to increase the mRNA synthesis of pro-collagen in mouse and human fibroblasts [159], and second, it acts as a cofactor of prolyl 3-hydroxylase, an enzyme that hydroxylates proline residues on the triple helix of collagen and stabilizes it [158,160]. First used to reproduce blood vessels, the self-assembly approach has also been applied to the reconstruction of various human tissues including skin, cardiac valves, adipose and urologic tissues, and, of course, the cornea [155,156,161,162,163,164,165].

**Figure 2:**
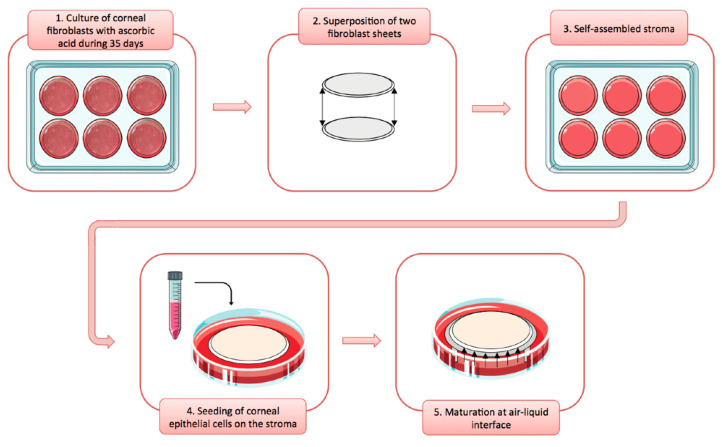
Schematic of the self-assembly procedure used to produce human tissue-engineered corneas (hTECs). Briefly, stromal fibroblasts are cultured with serum and ascorbic acid for 35 days. Two fibroblast sheets are then superimposed to form a reconstructed stroma on which corneal epithelial cells are seeded. The tissues are kept for 7 days under immersed conditions and 7 days at the air–liquid interface.

**Figure 3:**
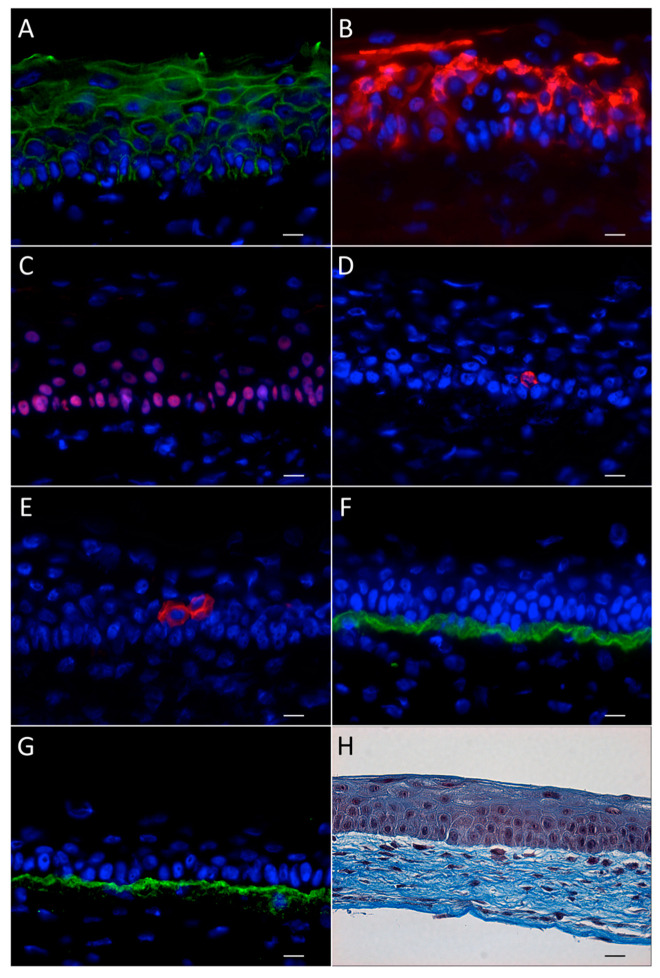
Characterization of the human tissue-engineered cornea. Immunofluorescence analysis of: (**A**) the epithelial barrier marker ZO-1, (**B**) the differentiation marker keratins K3/K12, (**C**) the epithelial cell marker p63, (**D**) the cornea epithelial stem cell marker ΔNp63α, and (**E**) K19, (**F**) the epithelial basement membrane components laminin V, and (**G**) collagen IV. (**H**) Nuclei were counterstained with Hoechst 33,258 reagent and appear in blue. Scale bar: 20 µm. Histology (Masson’s Trichrome staining) of the tissue-engineered cornea, showing an epithelium adhered to the self-assembled stromal matrix. The 5–6 epithelial cell layers differentiate during their upward migration. The scale bar in H equals 20 µm.

The procedure for reconstructing a tissue-engineered cornea by the self-assembly approach firstly consists in culturing hCFs with ascorbic acid for 35 days in order to stimulate the fibroblasts to secrete their own ECM. After 35 days, hCFs form thick cellular sheets that can be easily manipulated (Figure 2). Two fibroblast sheets are then superposed and cultured for one week, allowing the formation of a reconstructed corneal stroma [164]. hCECs are then seeded on the self-assembled stroma that is cultured for one week under immerged conditions and then lifted to the air–liquid interface for an additional seven days in order to promote the differentiation and stratification of the epithelium [166]. The reconstructed cornea, thus produced, is composed of a corneal stroma and a pluristratified epithelium (Figure 3) and can also be completed by the addition of a monolayer of corneal endothelial cells [167]. It is then possible to produce, using the self-assembly approach, a human tissue-engineered cornea histologically very similar to the native human cornea. Bilamellar hTEC could shortly prove to be an interesting in vitro model for studying corneal wound healing or drug–tissue interaction. In the future, it may be possible to use allogenic endothelial cells, since the rejection risk is low for these cells [55,168,169], or to grow autologous endothelial cells from induced pluripotent stem cells (iPS) in order to obtain a three-layer substitute [170]. This three-lamellar hTEC might eventually become the best, rejection-free alternative to repeat penetrating keratoplasty or to keratoprosthesis for patients with corneas at very high rejection risk.

#### 3.1.3. Characteristics and Advantages of the Human Tissue-Engineered Cornea

**Figure 4:**
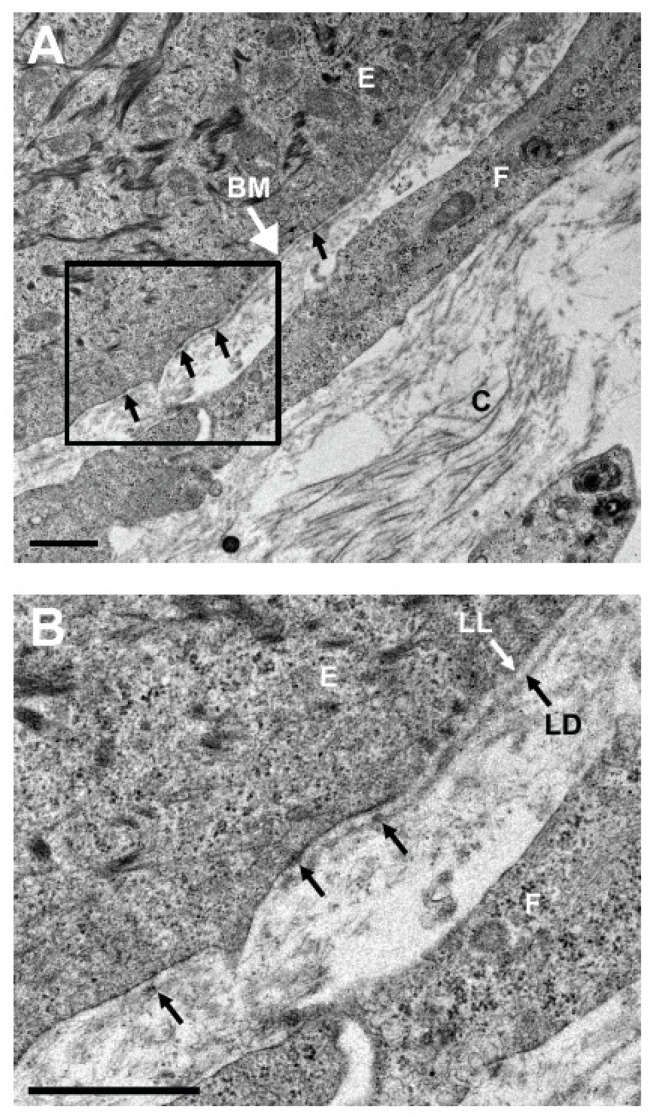
Transmission electron microscopic analysis of the hTEC. (**A**) Electron microscopic examination of the hTEC revealed the presence of an organized basement membrane (BM) with many hemidesmosomes (arrows) that attach basal corneal epithelial cells (E) to the underlying fibroblast sheet (F). A basement membrane is present at the junction between the epithelium and the stroma. Note the presence of the collagen fibers (C) surrounding the fibroblasts in the sheet. (**B**) Higher magnification that shows both the lamina lucida (LL) and lamina densa (LD), as well as the hemidesmosomes (arrows) are present in the BM. Scale bars: 1 µm.

The hTEC produced according to the self-assembly procedure is composed of a pluristratified epithelium made of four to six cell layers (Figure 3). While the basal epithelial cells are cuboidal with round nuclei, the superficial cell layers are rather characterized by the presence of non-keratinized flat squamous cells [164]. Keratin 3 and 12, the two main cytokeratins that serve as phenotypic markers of the corneal epithelium, can be found in some basal cells but mostly in the suprabasal cells of the reconstructed corneal epithelium [167]. This epithelium also lies on a well-organized basement membrane that forms a continuous lamina densa that is easily detectable in electronic microscopy [164,167,171] (Figure 4).

Laminin V and collagen VII are predominantly expressed in this basement membrane [164]. Hemidesmosomes, which connect basal cells to the underlying matrix, are also present at the epithelial–stromal junction [167,171]. Furthermore, both gene profiling and immunofluorescence analyses revealed that the reconstructed tissue has a pattern of expressed integrin receptors (that comprises the integrin subunits α2, α3, α5, α6, β1, and β4), which is very similar to that observed in the native cornea [171,172]. Interestingly, in the hTEC, the two integrin subunits α6 and β4 (both components of the hemidesmosomes) are relocated from the basal side of cells to the lateral and apical membranes during corneal wound healing, a phenomenon that also occurs in the native cornea [164].

The self-assembled stroma is made of hCFs entangled in their own ECM. Collagen type I is the predominant ECM component expressed, secreted, and assembled by the fibroblasts. Laminin, fibronectin, and other collagens, such as collagen types IV, V, VI, VII, and XII, all naturally present in the human cornea, can also be found in the reconstructed tissue [171,172]. Although usually absent from the native cornea, collagen type XIV and tenascin are also present in the hTEC [172,173]. One remarkable feature of the hTEC is that within the reconstructed stroma, collagen fibrils spontaneously adopt a structure similar to that found in vivo. Indeed, collagen fibrils in the self-assembled stroma can organize in lamellae, each superimposed at different angles, but all parallel with one another, an organization that is thought to confer its transparency to the human cornea [174]. Moreover, it has been shown that the reconstructed cornea mostly absorbs ultraviolet (UV) radiation around 265 nm, which appears to be close enough to 275 nm, corresponding to the UV absorption peak found in vivo [175].

The hTEC exhibits various advantages as a tissue substitute. First, it is devoid of any synthetic biomaterials, in order to better mimic the native cornea. It’s completely natural ECM, in many aspects similar to the native ECM, is a characteristic shared with other self-assembled constructs. The hTEC is made exclusively of untransformed human living fibroblasts and epithelial cells, which therefore makes it possible for the epithelial–mesenchymal interactions and cell–matrix interactions that are naturally present within the cornea to take place. Compared to immortalized cells, primary cells are also preferred since they are closer to native cells. hTECs produced by the self-assembly approach mimic many histological features of the native cornea, including a well-developed stratified epithelium and an adequate expression of basement membrane components and integrins, which makes them very similar to their in vivo counterparts. This tridimensional model also exhibits good translucency, an essential feature for a corneal substitute, and also good mechanical strength [174]. Furthermore, when mechanically wounded, the hTEC enhances migratory and proliferative cell properties, thereby promoting re-epithelialization and the restoration of a stratified epithelium, which is found to be very similar to the mechanisms occurring in the native cornea [164,173,176] and which makes them a relevant model for studying corneal wound-healing mechanisms.

#### 3.1.4. Recent Improvements Brought to the hTEC Model

The culture of hCECs has been developed using medium supplemented with growth factors and a very low concentration of cholera toxin [177]. Cholera toxin is a bacterial toxin secreted by *Vibrio cholerae* that acts as a mitogenic factor for hCECs by inducing intracellular accumulation of the second messenger cyclic adenosine monophosphate (cAMP) [178]. Since the demonstration of its efficacy, cholera toxin has been widely used as an additive for hCEC culture medium. The use of a feeder layer, in particular the murine i3T3 feeder layer, has also proven to be a key element for the optimal culture of a variety of cell types, including hCECs [71,177]. Indeed, previous studies have shown that a murine i3T3 feeder layer was effective in promoting hCEC proliferation and the maintenance of epithelial stem cells and progenitor cells [179,180]. Murine i3T3 are currently used as the most conventional feeder layer for the culture of hCECs. However, for clinical applications, both cholera toxin and i3T3 feeder cells present limitations for regulatory approval. Indeed, the handling of cholera toxin is regulated considering the risk it might pose to human health. The murine i3T3 feeder cells have been proven to secrete N-glycolylneuraminic acid (Neu5Gc), a non-human sialic acid, which could induce an immune response following transplantation [181,182]. Furthermore, this feeder layer could increase the risk of transmission of viruses (or viral fragments) from mice to humans, such as xenotropic murine leukemia virus (MLV)-related viruses [183]. For these reasons, we and others have recently improved the culture conditions of hCECs in order to decrease the potential risk of pathogen transmission from mice to humans and increase general safety for human health.

In the last few years, several groups have been working on the development of new feeder layers with the aim of finding an alternative to the use of i3T3. Human oral mucosal fibroblasts, umbilical cord-derived human unrestricted somatic stem cells, and human bone marrow-derived mesenchymal stem cells have been recently used as feeder layers for the co-culture of hCECs with promising outcomes [184,185,186,187,188]. Another group also succeeded in fabricating corneal epithelial cell sheets without a feeder layer by using a novel oxygen-controlled method [189]. We recently substituted i3T3 with a new irradiated human dermal fibroblast feeder layer [190]. This particular feeder layer has proven to be effective for culturing keratinocytes [191,192] and has even yielded higher growth rates and increased clonogenicity when compared with i3T3 [193]. We recently replaced cholera toxin with isoproterenol, another cAMP inducer whose use in keratinocyte culture goes back to 2005 [194]. Isoproterenol is a non-selective β adrenoreceptor agonist currently used for the treatment of bradycardia and heart block. It therefore has the advantage of being already approved for the clinic. While the measure of its relative effectiveness over cholera toxin has not reached a consensus yet [195,196], we recently demonstrated that isoproterenol was as effective as cholera toxin for stimulating and maintaining the proliferative potential of human skin keratinocytes upon cell passages, without, however, having any restrictions related to its use [193]. As for limbal epithelial cells, isoproterenol also proved to be more effective than cholera toxin for inducing cell proliferation, as well as for maintaining a smaller cell size [197]. Consequently, both iHFL and isoproterenol seem to be good alternatives to the use of i3T3 and cholera toxin, respectively.

In vivo, corneal endothelial cells are arrested in the G1 phase of the cell cycle. However, when appropriately stimulated, they are able to proliferate in vitro [198]. Consequently, culture media for corneal endothelial cells are traditionally supplemented with different growth factors and additives that act as mitogens, such as epidermal growth factor and chondroitin sulfate [199,200]. However, despite all the work done on the development of ideal growing conditions, corneal endothelial cells have a tendency to rapidly lose their endothelial phenotype upon passaging, evolving towards a fibroblastic-like phenotype. This phenomenon is now known as the endothelial-to-mesenchymal transition [201,202,203]. Among others, TGF-β1 has been shown to be a trigger for endothelial-to-mesenchymal transition in corneal endothelial cell cultures [202]. To address this problem, a novel dual media approach, which consists in culturing cells in two different media depending on whether cells are in the proliferation or the maturation phase, has emerged in the last few years. This approach allows the expansion of corneal endothelial cells over several passages while preventing the endothelial-to-mesenchymal transition [153]. Even so, it has been demonstrated that the addition of the growth factor TGF-β1 to corneal endothelial cells in their proliferating phase was deleterious for those cells and promoted a fibroblastic phenotype [202]. While seeking to further improve culture conditions, Beaulieu Leclerc et al. recently found that the addition of this growth factor was not always prejudicial. Indeed, the addition of TGF-β1 to corneal endothelial cells in their maturation phase proved to be beneficial for their phenotype and functionality [204]. It has been shown that the presence of TGF-β1 in the maturation medium could enhance endothelial morphology and also induce a better cytolocalization of Zonula occludens-1 (ZO-1), a tight junction-associated protein, at cell–cell interfaces [204]. Taking into account this new evidence concerning the contradictory effects of TGF-β1, a unique culture protocol for corneal endothelial cells was developed by this team, which consists in a dual media culture with a maturation phase in the presence of TGF-β1. Therefore, by exploiting this new approach, functional and high-quality endothelial cells can be obtained and used for the production of hTECs.

#### 3.1.5. What Are the ‘Missing Constituents?’

Although it is much closer to the native cornea than corneal epithelial cells grown as monolayers or on synthetic substrates can be, our human tissue-engineered cornea model can still be further improved. Indeed, some of its natural constituents, such as both nerve and immune cells, are absent from our hTECs. This section discusses improvements that can be brought to our corneal model to bring it much closer to the native human cornea.

##### Corneal Stromal Stem Cells

Corneal stromal stem cells (CSSCs) are present in the human corneal stroma and localized in the limbal palisades of Vogt, directly underlying the epithelial basement membrane where they maintain a close association with limbal epithelial stem cells [205,206,207,208,209,210]. Stem cells from the human stroma were initially identified as a side population, using the dye Hoechst 33,342 efflux [205,211]. CSSCs, like mesenchymal stem cells (MSCs), have been found to efflux fluorescent dyes, reducing their fluorescence and thus allowing their identification by flow cytometry as a side population. CSSCs could be expanded clonally through 100 cumulative population doublings. Genes specifically expressed by these cells include the MSC genes ABCG2, BMI1, and CXCR4, as well as genes present in early corneal development, such as PAX6 and Six2 [205,212]. Like mesenchymal stem cells (MSCs), CSSCs are multipotent, a key identifier of stem cells.

When CSSCs are grown in serum-free medium supplemented with insulin and ascorbate, they upregulate expression of the keratocyte-specific markers ALDH3A1, CXADR, PTDGS, and PDK4 [205]. These results are very interesting when looking at improving the hTEC model. Indeed, expanding stem cells in culture and then differentiating them into keratocytes could provide useful cells for tissue engineering of the cornea and for cell-based therapeutic applications. Usually, the in vitro expansion of adult keratocytes typically leads to their transition towards a fibroblastic morphology, which produces a scar-like ECM rather than the specialized ECM required for corneal transparency [213]. It has been shown that when CSSCs and hCFs are grown under equivalent culture conditions, secretion of collagen types I, V, and VI, the major protein components of the corneal stromal tissue, was much less abundant in the hCF-secreted constructs than in those produced by hCSSCs. Furthermore, most studies have reported that the ECM secreted by hCFs is lacking the cornea-specific keratan sulfate proteoglycans, keratocan and lumican, that are critical to the regulation of inter-fibril spacing and thus for corneal transparency [214,215,216,217,218,219,220].

When CSSCs are removed from their substratum and cultured as a pellet in low-mitogen, ascorbate-containing media, a more complete keratocyte gene expression pattern was observed and significant amounts of ECM were deposited, some with tracts of aligned collagen fibrils, similar to what is observed in the stroma in vivo [212]. Like hCFs, when CSSCs are cultured on a substratum of parallel aligned polymeric nanofibers, they produce layers of highly parallel collagen fibers with packing and fibril diameter close to that of human stromal lamellae [174,221,222,223], thereby demonstrating a role for topographical cues in guiding the organization of corneal tissue. Furthermore, the addition of fibroblast growth factor-2 (FGF-2) and transforming growth factor- β3 (TGF-β3) enhanced the effects of the geographical cues, allowing the generation of a corneal stroma-like tissue made up of multilayered lamellae with orthogonally oriented collagen fibrils and abundant in cornea-specific proteins and corneal keratan sulfate proteoglycans (keratocan, lumican, decorin) [220].

By comparison, hCF-secreted ECM exhibits characteristics of human corneal scar tissue, in which collagen fibrils lack the hierarchical organization of human corneal stromal, inter-fibril spacing is irregular, and keratocan expression is lacking [220]. In accordance with a repair ECM protein profile, the stromal substitutes engineered using fibroblasts have been shown to express fibronectin [172,224] and lack the expression of keratocan (a keratocyte phenotypic marker) [225,226]. However, the stromal substitutes expressed very low levels of type III collagen and α-SMA, suggesting that the ECM was deposited by fibroblasts and not by myofibroblasts [224].

##### Innervation

Somatosensory innervation of the cornea serves to identify changes in environmental stimuli at the ocular surface, thereby promoting the barrier function in order to protect the eye against injury or infection. As the most densely innervated tissue in the body, the cornea contains intraepithelial nerve fibers that originate from the sub-basal nerves, giving rise to an extreme sensitivity of the tissue [227,228]. Structurally, nerves enter the cornea radially from the periphery to form the sub-basal nerve plexus with intraepithelial nerve fibers. This sensory presence is prominent at the cornea-scleral rim at 200 μm from the ocular surface with additional bundles distributed from 50 to 500 μm deep within the stroma [228]. The means by which the cornea is able to retain homeostasis, transparency, structural rigidity, and regeneration throughout a lifetime relies on this interplay between the peripheral nervous system and resident cells within the tissue (epithelial, stromal, and immune cells) via secreted factors, like exosomes (recent work highlighting their potential mediators of epithelial–stromal interactions) [229]. Damage that affects peripheral nerve functionality may lead to deleterious effects on the integrity of the corneal surface [230,231,232,233].

The rapid degeneration of nerve endings in the cadaveric cornea limits long-term studies of ocular irritation since nociceptive functionality is lacking [234]. Furthermore, the nerve loss of the excised cornea post-transplantation increases the difficulty of attempting to study nerve structure in the human cornea. So, there is a need to develop functional human tissue models that can predict ocular damage and pain using in vitro-based systems to increase throughput and minimize ex vivo model and animal use. Corneal tissue models that fully mimic the anatomy, mechanical properties, and cellular components of the human cornea would provide useful systems for the study of cellular interactions, corneal diseases, and corneal wound healing. Furthermore, these in vitro models with functional innervation would be sophisticated tools for studying ocular nociception.

There are currently few 3D in vitro corneal models available which contain corneal innervation along with the physical and structural properties of the corneal tissue. Recently, a corneal tissue model comprising a stroma, an epithelium, and innervation was generated [111]. A thin silk protein film served as scaffolding to support the corneal stromal layer assembly and stratified epithelium formation [235,236]. A surrounding silk porous sponge has been used to grow chicken dorsal root ganglions (DRG) or human-induced neural stem cells in order to generate cortical neurons cultured in a 3D environment [111,237]. In order to guide axons towards the top of the scaffolds, a collagen hydrogel containing nerve growth factor (NGF) was cast on top of the film stack. Further advances include the application of a self-assembled stromal model. This relies on ECM production by corneal fibroblasts and the addition of a bone-marrow-derived neuroblastoma cell line (SH-SY5Y), differentiated into a neuronal lineage [238]. Furthermore, the ECM plays a fundamental role in regulating the growth and functionality of peripheral nerves [239,240]. These studies emphasize the need to implement 3D model approaches in conjunction with in vivo models in the study of pain mechanisms.

Collectively, the application of these human-based in vitro models will provide an opportunity to improve our cornea model. In fact, it would be possible to seed DRG or human-induced neural stem cells at the periphery of hCFs or CSSCs sheets. Then, these sheets would serve as supports for the successive stacking of other cellular sheets. After the reconstruction of the corneal stroma and the seeding of hCECs, NGF would be cast on top to guide axon migration.

##### Immune Cells

The immune system plays a fundamental role in protecting the complex and fragile structures of the surface of the eye. The cornea and conjunctiva are adjacent mucous membranes, subjected to the same stresses but characterized by totally different immunological responses: while the conjunctiva is hyperreactive in the immuno-inflammatory mode, the cornea is much less reactive and presents an inhibition of inflammatory reactions [241]. This is called the immune privilege of the cornea [242]. Although the immuno-inflammatory response is a formidable weapon, it also sometimes has undesirable effects, for example by damaging the surrounding healthy structures [243]. In contrast to other tissues including the skin, the cornea usually does not respond with the induction of blood and/or lymphatic vessels to minor injuries and angiogenic stimuli. Such reactions would interfere with its transparency and result in loss of vision, a characteristic commonly referred to as corneal angiogenic privilege [244]. Therefore, the corneal angiogenic and immune privileges, which are two closely linked but distinct processes, are actively maintained and ensure corneal transparency.

Immune cells are found in the corneal tissue. Leukocytes, for instance, are located mostly in the corneal periphery, but also in the epicentral and central cornea [245,246]. Furthermore, CD45+ cells are distributed throughout the entire depth of the stroma [246] where they are localized primarily in the posterior stroma and uniformly distributed in the periphery and center of the cornea [245]. CD11b-positive macrophages were shown to make up about 50% of the resident corneal leukocytes [246]. Langerhans cells, a type of dendritic cell, are found in the limbal epithelium, and both at the periphery and in the center of the cornea [247]. The slow-cycling cells that have been identified in the limbal basal epithelium are putative precursors of Langerhans cells [248]. Similar to the distribution of Langerhans cells, stromal dendritic cells are found in the periphery and center of the anterior stroma, with the central cells [245,249]. LysM-positive neutrophils are located around the limbal vessels in the periphery but are not found in the central cornea [250]. Similarly, mast cells are found in the corneal limbus and conjunctival parenchyma, but not in the central cornea. Although in earlier reports CD3^+^ T cells had been reported to be absent from the cornea [246], resident CD4^+^ and CD8^+^ T cells have now been identified in the central and peripheral region of native corneas [251].

In general, immune cell recruitment after corneal injury is mediated by proinflammatory cytokines released from epithelial cells and keratocytes at the injured site. Il-1, Il-6, and TNFα have been shown to be important mediators of that process [252,253,254,255,256]. Being attracted by these and several other cytokines, recruited leukocytes from the limbal blood vessels enter the stroma and migrate towards the wound site [257]. Neutrophils are the first cells to infiltrate the cornea after injury: they can be detected up to 48 h after epithelial abrasion or injury [258]. Shortly, macrophages extravasate from the limbal vessels, infiltrate the stroma from superficial to deeper layers, and migrate towards the center of the cornea [257]. Macrophages remove debris and apoptotic cells at the wound site but have also been shown to be essential mediators of angiogenesis after severe and prolonged corneal injury [259,260,261,262]. Macrophages also take part in corneal wound closure by secreting TGF-β to promote the differentiation of fibroblasts into myofibroblasts [263,264]. Centripetal migration of Langerhans cells, from the limbal basal epithelium, into the central cornea was shown to be mediated by IL-1, TNF, and CCR5 signaling [265,266]. The rise in Langerhans cell number at the corneal surface also increases the antigen-presenting capacity of the cornea, which is important in order to adequately respond to foreign antigens that might have been introduced through the wound [266,267]. Natural killer cells, which are rare in the native intact cornea, were also shown to accumulate around the limbal vessels and to infiltrate the corneal stroma from the periphery to the center [268]. These cells promote epithelial wound closure and the regeneration of corneal nerves and orchestrate the corneal inflammatory response [268,269]. γδ T cells were shown to promote wound healing and influence neutrophil and platelet numbers in corneal inflammation [270,271,272].

The inclusion of inflammatory cell types may also be an interesting development in cornea models, given the contributory role of inflammation in wound healing. This area remains relatively unexplored in corneal tissue models but may be important in studying ocular infection and inflammatory pain in vitro. The addition of resident and invading immune cells may be useful for the study of nerve–immune cell crosstalk or epithelial–immune cell crosstalk in a system readily adaptable to temporal studies. Unlike the cornea, several research groups have successfully incorporated immune cells into their tissue-engineered skin models [273,274,275,276,277]. The addition of Langerhans cells [273,276,277,278,279], dendritic cells [273,274], macrophages [280], and T cells [281] was made possible in order to generate the much more complete models needed to study processes (disease, scarring, infection) involving inflammation. The cells used to generate immune-competent models are usually partially or fully sourced from donor skin samples and peripheral blood [273,277,280,281]. As for the skin [282], the addition of immune cells such as macrophages, Langerhans cells, or dendritic cells to the corneal stromal sheet would surely prove to be particularly interesting in order to generate an immunocompetent hTEC.

## 4. The Future of the hTEC: Potential Applications and Uses

The ECM and cellular composition of hTEC, which are relatively similar to that of the native cornea, make it an excellent model for various research and clinical applications. Indeed, hTECs can be used to study various physiological processes at both the cellular and molecular levels, such as the mechanistics of corneal wound closure. The hTEC can also be used for in vitro designing and testing of different drugs or procedures that may prove of interest for the treatment of corneal pathologies such as LSCD. This can range from a simple topical treatment, to tissue transplantation in the patient, to gene therapy.

### 4.1. A Graftable Alternative in the Treatment of LSCD

The corneal epithelium is a self-renewing tissue that is maintained by stem cells localized in the peripheral limbus. The destruction of the limbal stem cell niche results in LSCD [240]. Congenital or acquired LSCD impairs corneal epithelium renewal, resulting in progressive opacification, chronic ulceration, conjunctivalization, and neovascularization with blindness, disfigurement, and occasionally discomfort as a result. If the damage extends to the anterior corneal stroma, fibroblastic cells derived from stromal keratocytes may produce long-lasting opaque scar tissue [283,284]. In many cases, stromal opacities and neovascularization improve once the corneal epithelial phenotype is restored by limbal stem cells and corneal epithelium regeneration. If it does not, additional surgery such as penetrating or lamellar keratoplasty can be tempted to recover corneal transparency.

The use of corneal epithelial sheets for the treatment of LSCD has represented significant progress for the management and outcome of this condition. The knowledge of limbal stem cells, the improvement of culture methods, and the development of surgical procedures adapted to corneal epithelial sheets have defeated such a challenging disease as LSCD [285]. Indeed, twenty years of clinical studies have resulted in 70–80% success rates for the treatment of LSCD with autologous corneal epithelial sheets [71,73,285,286,287,288,289,290,291,292,293,294,295,296]. The effectiveness of CLET using HAMs or fibrin gel support for transplantation is now well established [73,92,297,298,299,300]. However, the underlying stroma with large scars will maintain a certain opacity of the cornea. Surgical replacement of the cornea is the primary approach for the restoration of the patient’s vision. In a study by Pellegrini et al. conducted on 152 patients treated for unilateral LSCD with CLET, 56 patients were subjected to anterior lamellar or penetrating keratoplasty to improve the vision of eyes with stromal scarring [286]. For these 56 patients, limbal stem cells grafted previously proved successful for the regeneration of a corneal epithelium.

In the presence of a healthy contralateral eye, unilateral LSCD is usually addressed with techniques such as CLAu, SLET, or CLET. CLAu and SLET have the advantage of being more easily accessible and less costly, since the need for a specialized laboratory is not necessary for their preparation. When both eyes are compromised with significant LSCD, CLAu or SLET are not the procedures of choice because they bear the risk of badly depleting limbal stem cells from the donor eye, worsening the clinical situation. Under such circumstances, limbal stem cells allografts obtained from living related or cadaveric donor eyes is the preferred limbal stem cells replacement technique [301]. The major disadvantage for allogenic transplants is the need for long term systemic immunosuppression to prevent graft rejection. On the other hand, CLET has been shown in our laboratory to have the potential to generate, from one small limbal sample, enough autologous corneal epithelial and stem cells to treat more than one limbal stem cells’ deficient cornea. Moreover, the cultivated cells can be frozen for long periods of time and used whenever it might become necessary. We recently successfully treated a patient with a decompensated first CLET due to an inflammatory ocular surface disease by using the frozen expanded cells harvested five years before [296].

Eyes with unilateral severe LSCD and badly scarred stroma are usually taken care of with a two-step procedure: a corneal stem cell transplantation (CLAu, SLET, CLET) followed, at a later time, by a Lamellar or a Penetrating Keratoplasty. The use of a bilamellar hTEC [296] would eventually enable a one-step technique, possibly leading to a less expensive overall procedure and a faster recovery. Long-term stability of the grafted stem cells in those circumstances and the viability of the reconstructed stroma would have to be investigated further before such a clinical application is attempted.

The demonstration that ECM sheets produced by stromal cells are biocompatible in vivo when transplanted in feline corneas has been reported [302]. These stromal substitutes composed of ECM sheets produced by corneal stromal fibroblasts were inserted in a lamellar stromal pocket and followed for 4 months. The stromal substitutes were well-tolerated, showing a normal fibrillar stromal ultrastructure and progressive reinnervation. These stromal substitutes showed low immunogenicity and high biocompatibility in vivo. Since the fibroblast phenotype is reversible in vivo, it is hypothesized that the fibroblasts in the stromal substitutes engineered using serum would revert into keratocyte-like cells once implanted. There are other examples of tolerance reported for these cells. For instance, stem cells isolated from human corneal stromas and injected into mouse corneal stromas remained viable for 10 weeks without eliciting an immune response. The injected cells deposited lumican and keratocan, suggesting that stem cells differentiated into keratocytes in vivo [303]. Furthermore, using limbal biopsy-derived stromal cells embedded in fibrin gels and engrafted into mouse corneal wounds, these authors showed that their limbal stem cells differentiated into keratocytes in vivo and induced regeneration of the damaged stromal tissue without scarring [303]. In fact, the transplanted stromal cells did not only survive and differentiate in vivo, but they were also functional after grafting in mice. This recent discovery opens the door to the development of autologous treatment for stromal scarring. Further research is necessary to evaluate whether human corneal stromal substitutes composed of assembled insular sheets secreted by hCFs or CSSCs cultured with ascorbic acid could be effective for treating stromal scarring. Corneas with anterior scarring that contain a competent endothelium are often treated by partial-thickness anterior lamellar keratoplasty. The efficacy of this process is similar to that of penetrating keratoplasty, but it is considered safer [304]. Unfortunately, many of these grafts are lost within 3–5 years [305]. The grafting of partial-thickness stromal substitutes for diseased anterior stromas could become an alternative in the case of failure of a penetrating keratoplasty.

Full-thickness stromal substitutes would be used for pathologic corneas that are not amenable to lamellar transplantation because of the extent of the disease or scar tissue. They are more challenging to develop, as several criteria need to be met. Indeed, these substitutes must faithfully mimic the main functions of a normal cornea, namely transparency, refraction, and ocular protection. Corneal equivalents must have corneal stromal deturgescence in order to maintain optical transparency, which requires a functional endothelium. Full-thickness 3-layer tissue-engineered corneas (epithelium-stroma-endothelium) have been produced using primary cells and immortalized cells [306,307]. These full-thickness stromal substitutes would require more rigorous testing before they could be used in clinical trials. It is obvious that the development of an autologous, tissue-engineered, three-layered corneal tissue would be an important advancement for the treatment of corneal pathologies. Currently, the only devices used to replace a penetrating graft in human subjects are the keratoprostheses (Section 1.2).

### 4.2. A Model for the Study of Corneal Wound Healing

Corneal wound healing is a complex event involving many processes such as cell death, proliferation, migration, adhesion, and differentiation [308]. During these steps, the composition of the ECM is continually modified to allow proper re-epithelialization, epithelial cell migration, and differentiation [309,310]. The ECM is a non-cellular network of proteins and polysaccharides to which cells adhere through cell–matrix interactions [311]. Alterations of the ECM occurring during corneal wound healing include the massive transitory secretion of fibronectin combined with a reduction in the secretion of collagens and laminins [312,313,314]. This ECM remodeling is required to allow the fast migration of corneal epithelial cells, in order to rapidly cover the damaged area. Changes in the composition of the ECM are perceived by integrins, a family of membrane-anchored receptors that recognize the different components of the ECM as their ligands [172,315]. The downstream cascades of mediators activated by these cell–matrix interactions then lead to the transcription of genes involved in wound healing [316].

**Figure 5:**
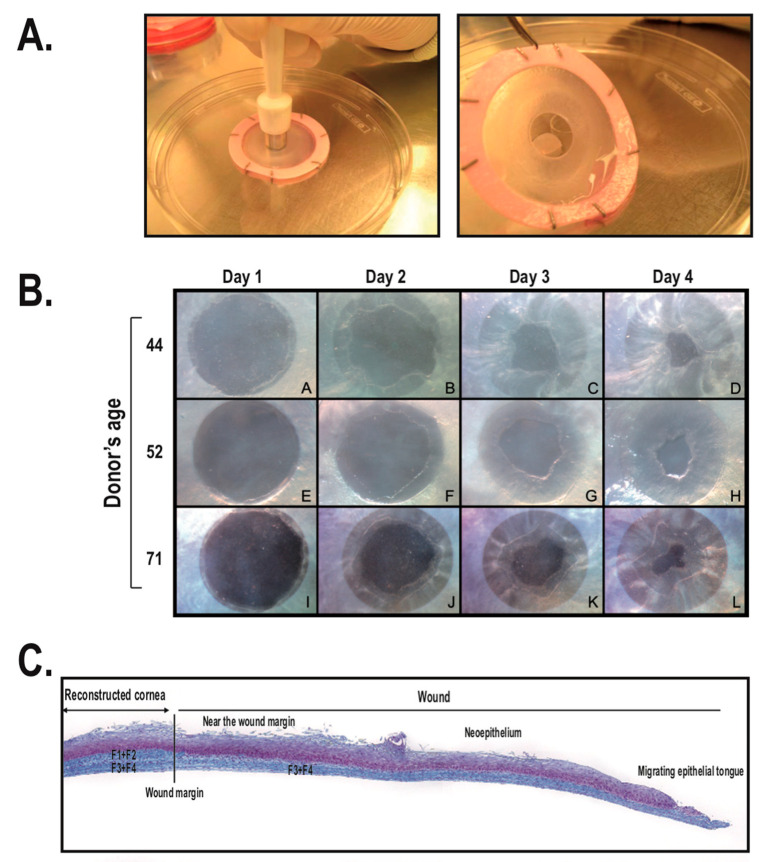
Production of wounds on human tissue-engineered corneas. (**A**) The reconstructed cornea is wounded using an 8 mm biopsy punch. (**B**) Closure of wounds on tissue-engineered corneas produced using HCECs isolated from the eyes of three different donors (44, 52, and 71 years old). Closure of the wounded epithelia was followed from 1 to 4 days after corneal injury. (**C**) Composite image showing a complete view of the wounded tissue-engineered human cornea 3 days following corneal damage (sections were stained with Masson’s trichrome; cells are pink and collagen is bluish). The wound margin created by the biopsy punch is indicated. F1 + F2: initial fibroblast sheets present in the reconstructed cornea prior to wounding. F3 + F4: supplementary fibroblast sheets added following wounding of the tissue-engineered corneas (Figure adapted from Reference [173] with the permission of the journal Biomaterials).

Several models of wound healing have been developed in order to investigate the corneal mechanisms of re-epithelialization and to screen for growth factors susceptible to stimulate an adequate healing response [317,318,319,320,321,322,323,324,325]. Although very convenient because of their ease of use, cell monolayers in in vitro models suffer, however, from the lack of epithelial–mesenchymal interactions and a limited epithelial thickness. In addition, studies of corneal wound healing in animal models are very expensive and inter-individual variability among animals is inherent to in vivo experiments. Progress in tissue engineering has resulted in the development of hTECs designed to mimic their in vivo counterpart in terms of cell phenotype and tissue architecture (see Ref. [326] for an extensive review). As detailed in Section 3 above, we have succeeded in producing hTECs that show appropriate histology and expression of basement membrane components and integrins [164,167,175,306,327,328,329,330]. Besides being devoid of any synthetic materials, corneas tissue-engineered by our self-assembly approach exhibit a well-developed stratified epithelium that expresses differentiation markers (such as keratin 3) and that comprises a stroma and a well-organized basement membrane (Figure 3 and Figure 4). When mechanically damaged (using a biopsy punch), this fully human hTEC, produced from living hCFs and untransformed hCECs, mimics many aspects of the re-epithelialization process, including cell migration and proliferation, and the restoration of a stratified epithelium [164,175] (Figure 5).

Our recent use of the hTEC as a biomaterial for the study of both the cellular and molecular mechanisms of wound healing revealed important alterations in the pattern of genes expressed in response to wound healing [173]. Indeed, the expression of many metalloproteinase (MMPs)-encoding genes was shown by microarray and qPCR analyses to increase in the migrating epithelium of wounded corneas. In addition, the expression of MMPs by hCECs was affected both by the hCFs and the collagen-enriched ECM they produce. Most interestingly, the results from mass spectrometry analyses provided evidence that a fully stratified epithelium is required for proper synthesis and organization of the ECM to which the epithelial cells adhere [173].

**Figure 6:**
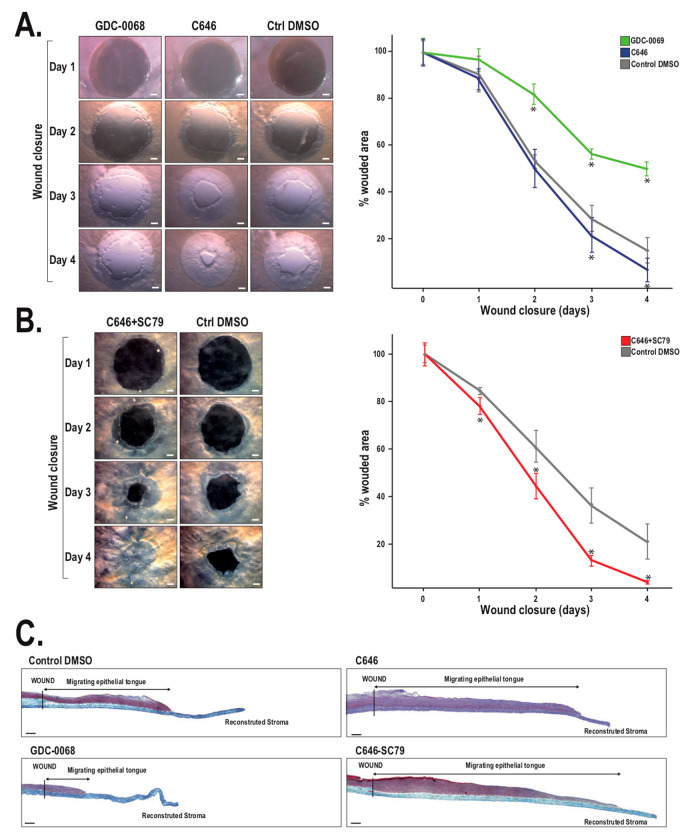
Impact of GDC-0068, C646, and SC79 on wound closure of human TECs. (**A**) Human TECs (one out of four representative hTECs is shown for each condition) were wounded and maintained in culture medium supplemented with GDC-0068 or C646 (**left**). Wound surfaces remaining for each condition were determined at each day and plotted on the graph (**right**). Control wounded hTECs were exposed solely to the vehicle (DMSO). Scale bars: 1 mm. (**B**) Wounded hTECs were incubated with both C646 and SC79 (**left**); one out of six representative hTECs is shown for each condition). Wound surfaces remaining were determined at each day and plotted on the graph (**right**). Control wounded hTECs were exposed solely to the vehicle (DMSO). Scale bars: 1 mm. (**C**) Composite images showing a complete histological view of wounded TECs grown in the presence of GDC0068, C646, or both the combination of C646 and SC79 at 4 days following corneal damage (sections were stained with Masson trichrome; cells are pink, and collagen is bluish). The wound margin created by the biopsy punch is indicated. Scale bar: 100 μm (Figure adapted from Reference [171] with the permission of the journal ACTA Biomaterialia).

The major signal transduction pathways activated by the integrins include the Janus kinase/signal transducers and activators of transcription (JAK/STAT) [331], mitogen-activated protein kinase (MAPK) [332], and phosphoinositide-3-kinases/protein kinase B (PI3K/AKT) pathways [333,334]. More recently, the β2 integrin subunit from the immune cells was also identified as an upstream regulator of the actin-regulated myocardin-related transcription factor A/serum response factor (MRTF-A/SRF) pathway in response to external cell stimuli that initiate F-actin polymerization downstream of RhoA activation [335]. However, in corneal wound healing, little is known about which pathway contributes the most to the healing process. The hTEC was recently used as a model for studying the signal transduction pathways that participate in corneal wound healing. By exploiting both gene profiling and activated kinase arrays, the occurrence of important alterations in the level of expression and activation of a few mediators from the PI3K/Akt and C-AMP responsive element-binding protein (CREB) pathways in response to the ECM remodeling taking place during wound healing of damaged hTECs could be demonstrated [171]. The pharmacological inhibition of CREB with C646 considerably accelerated wound closure compared with controls. This process was further accelerated when both C646 and SC79, an agonist of Akt, were added together to wounded hTECs (Figure 6), suggesting that proper corneal wound healing requires the activation of Akt together with the inhibition of CREB and that in vitro wound re-epithelialization can be improved by the use of pharmacological inhibitors (such as C646) or agonists (such as SC79) of these mediators.

**Figure 7:**
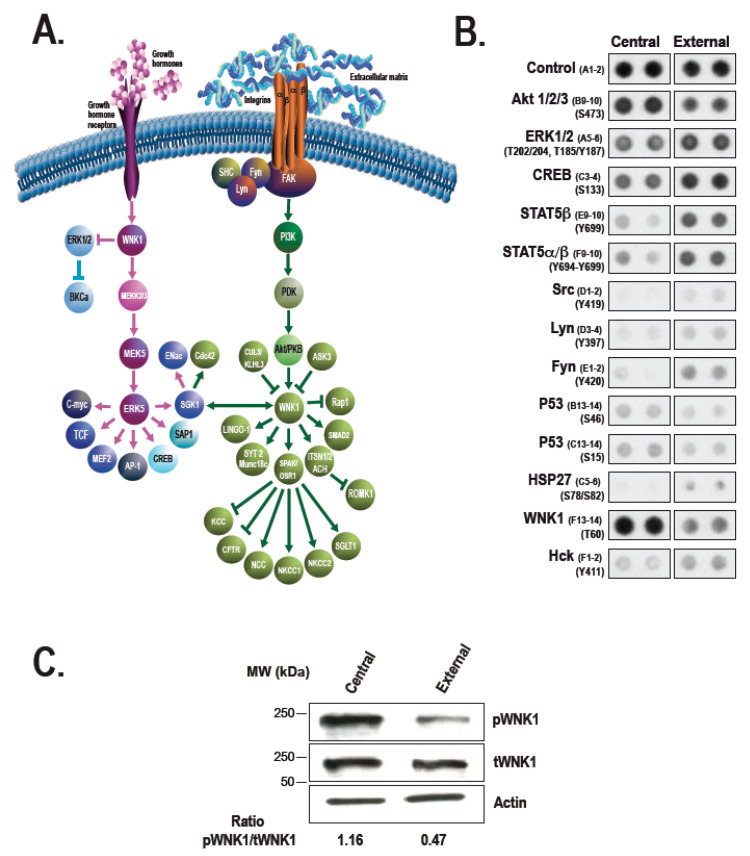
Kinase profiling analysis during corneal wound healing. (**A**) Major protein mediators from the WNK1 signal transduction pathways. (**B**) Cell lysates isolated from the central and external areas of hTECs assembled using hCEC-44, hCEC-52, and hCEC-71 were pooled together and used for the detection of activated kinases with the *Human Phospho-Kinase Array* from R&D Systems. Kinases and mediators identified as being differentially phosphorylated between the central (wounded) and external (unwounded) areas of hTECs are identified. (**C**) Cell lysates from the central and external areas of wounded hTECs were analyzed by immunoblotting to confirm the phosphokinase array results for the mediator WNK1. Actin was used as the loading control (Figure adapted from Reference [336] with the permission of Journal of Tissue Engineering and Regenerative Medicine).

In a recent attempt to further characterize the mechanistic details of the signal transduction pathways activated during corneal wound healing, we have shown that phosphorylation-mediated activation of the WNK1 kinase was one particularly important event occurring during hTEC wound healing [336] (Figure 7). WNK1 is the founding member of a family that comprises four evolutionarily conserved serine–threonine kinases (WNK1, WNK2, WNK3, and WNK4) that share 85% homology over their kinase domains [337]. The activation by phosphorylation of WNK kinases allows them to respond to changes in intracellular chloride concentration [Cl^−^] and tonicity [338,339]. Whereas the expression of WNK2 and WNK3 is usually restricted to the kidney, that of WNK1 is ubiquitous (reviewed in Ref. [340]). In agreement with these results, unwounded hTECs were found to significantly express only the WNK1 mRNA transcript [336]. The especially important contribution of this kinase to the wound healing process was demonstrated by the inability of wounded hTEC to properly heal upon pharmacological inhibition of WNK1 by WNK463, a process that is likely mediated by the WNK1 downstream target mediators SPAK/OSR1 [336]. How, then, might the activation of the WNK1/SPAK/OSR1 pathway contribute to wound healing? The answer to this puzzling question might come from the recent demonstration that activated WNK1 is a key participant in ensuring a proper balance between adhesion and migration in T cells. Indeed, the activation of WNK1 through the PI3K/Akt pathway was found to negatively regulate integrin-dependent T-cell adhesion while improving their migratory properties [341]. It is the activation of the WNK1-downstream pathway OSR1-SPAK-NKCC1 that has been demonstrated to regulate cell migration, likely through a mechanism involving ion transport across the cell membrane. This interesting hypothesis suggests that polarization occurs at the leading edge through a *Slc12a* Na-K-Cl co-transporter-dependent uptake of ions and water, whereas these are released at the trailing edge of the migrating cells, a process that would cause cell movement and described as the ‘osmotic engine model’ [342,343]. In summary, activation of the WNK1-SPAK/OSR1 pathway appears to be required to ensure proper wound closure of wounded hTECs. The activation of this signal transduction pathway during corneal wound healing raises the interesting possibility that ions and water transport in addition to actin reorganization might contribute to the proliferative and adhesive/migratory properties of the corneal epithelial cells located near the wounded area.

### 4.3. hTECs and Nanotechnologies: A Model for the Development of a New Drug Delivery System

Nanotechnologies are increasingly associated with multiple systems for biomedical applications, including tissue engineering for regenerative medicine [344]. Indeed, these molecular-level technologies enable the modification and improvement of system properties, such as their interactions with cells, their topology via nanofabrication, and the controlled release of drugs or active substances [345]. For example, nanotechnology approaches in tissue engineering are currently being developed for peripheral nerve regeneration [346], in which different scaffolds, based on natural, synthetic, or semi-synthetic materials, are combined with nanotechnology to improve the targeting, guidance, or release of active substances, such as neurotrophic factors. Similar strategies are being developed for different organs such as the bladder [347], blood vessels [348], heart [349], skin [350], and bone [351,352], as well as for craniofacial and dental materials [353]. These recent advances in the integration of nanotechnologies in tissue engineering could dramatically improve tissue properties, and thus have a significant impact on their clinical developments.

In order to reduce the number of animal assays, as recently announced and targeted by the American Food and Drug Administration (FDA) [354], the use of 3D models as preclinical models is becoming increasingly popular for cytotoxic assays and pharmacokinetic studies [355]. The active substances are often hydrophobic and unstable, and their formulation must be optimized to allow their biocompatibility as well as better penetration and efficiency, depending on the route of administration. Tissue engineering is therefore an excellent system for testing a large number of experimental conditions.

A hTEC platform was recently developed to assess drug absorption in the anterior eye [356]. This system, named the Dynamic Micro Tissue Engineering System (DynaMiTES), was tested with a human hemicornea construct [357] in the presence of sodium fluorescein and different concentrations of benzalkonium chloride, a controversial preservative [358]. This proof of concept represents a real advance for the use of tissue-engineered corneas for pharmacokinetic studies of promising new drugs. Furthermore, hTECs are progressively completed with different aspects, such as innervation [359]. This new functionality allows the exploration of different drugs for their influence on the corneal innervation.

Another commercially available model of hTEC (SkinEthic Laboratories, Nice, France) was used to assess the eye-irritating potential of chemicals through viability assays [360]. A bovine tissue-engineered cornea was also used for ophthalmic permeation studies of pilocarpine, currently used for glaucoma treatment, in order to evaluate the influence of different formulations [361]. Furthermore, drug permeation studies were performed on hTECs and compared to porcine corneal constructs, excised porcine corneas, and human donor corneas [134,362]. hTECs could therefore play a key role in preclinical testing during the development of drugs and drug delivery systems, thus reducing the use of animal models.

**Figure 8:**
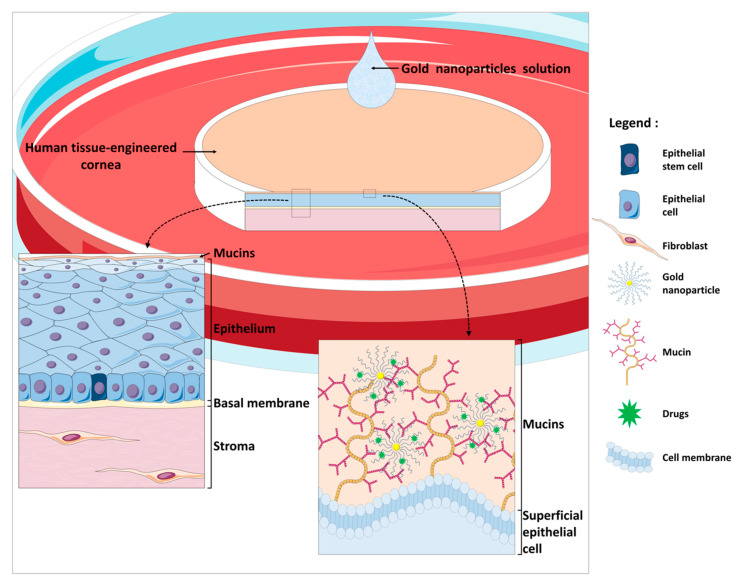
The hTEC as a model for the development of a gold nanoparticle drug delivery system. Illustration of the use of the hTEC as a platform for the development of drug delivery systems. **Left** insert: layers composing the hTEC (stroma, basal membrane, epithelium, and mucins) for the assessment of drug absorption, controlled release of drugs, permeation studies, bio-distribution, cytotoxic assays, efficacy assays, and pharmacokinetic studies. **Right** insert: Mucosal layer in the tear film, composed of mucins that can interact with mucoadhesive molecules such as the gold nanoparticles, as shown here.

As stated in Section 4.2, we recently demonstrated the clinical interest of pharmacologically altering the activation status of signal transduction mediators, such as CREB and AKT, which also play a critical role during corneal wound healing in vitro [171]. However, the low solubility and stability of these compounds could benefit from nanotechnologies to improve their properties. For example, mucoadhesive ultra-stable gold nanoparticles are currently being developed in order to modulate their formulation and achieve better efficiency (unpublished results) [363,364,365,366,367,368]. In this case, hTECs could thus serve as a platform for performing preclinical tests such as cytotoxicity, biodistribution, drug release, and efficiency assays (Figure 8).

## 5. Future Directions and Conclusions

The development of full-thickness stromal substitutes for pathologic corneas that are not amenable to lamellar transplantation because of the extent of the disease or scar tissue truly represents a promising avenue despite the fact their development proves to be more challenging. Full-thickness three-layer tissue-engineered corneas (epithelium-stroma-endothelium) have been produced using primary cells and immortalized cells [306,307]. These full-thickness stromal substitutes require more rigorous testing before they could be used in clinical trials. It is obvious that the development of an autologous, tissue-engineered, three-layered corneal tissue would be an important advancement for the treatment of corneal pathologies such as LSCD. Indeed, despite the inherent laboratory cost for the preparation of CLET, this technique holds great potential with respect to the treatment of bilateral LSCD when a minimal number of limbal stem cells is still available in at least one of the diseased corneas. It is conceivable that with a single harvest of limbal cells, we might soon be able to treat both corneas without having to depend on techniques (such as limbal stem cells allografts) requiring long-term systemic immunosuppression and their potential severe side effects [16]. We are currently starting a new clinical research protocol to address this issue.

Recent advancements in the field of gene therapy have led to the development of the *clustered regularly interspaced short palindromic repeats* (CRISPR) *-associated protein 9* (CRISPR-Cas9) technology. This technique offers great advantages compared to other gene editing procedures such as RNA interference (RNAi), as it is quite simple and highly efficient [369]. It relies on the enzymatic activity of the Cas9 nuclease, an RNA-guided DNA endonuclease from *Streptococcus pyogenes* that induces a double-strand break at a very specific DNA sequence. In most cases, the DNA is repaired by Non-Homologous End Joining (NHEJ) and the resulting protein is truncated, and therefore not functional [370]. Besides this Cas9 gene knockout property, more sophisticated CRISPR methods now allow the alteration of the expression of any given gene either positively (gene activation) or negatively (gene repression), which is of particular interest in some diseases. In those cases, the Cas9 enzyme is deactivated (dCas9) and coupled with enhancers (such as VP16) [371] or repressors of chromatin compaction (such as the Krüppel associated box domain (KRAB)) [372]. This technology is growing at a very rapid pace. It has been proven to be secure and efficient enough to be used for gene therapy. At the moment, there are 30 different clinical trials being conducted worldwide that use the CRISPR-Cas9 technology as a therapeutic gene editing agent (https://clinicaltrials.gov/ct2/results?cond=&term=CRISPR&cntry=&state=&city=&dist=). While these clinical trials mostly focus on treating several types of cancers, only one affects the eye, the Leber Congenital Amaurosis, a retinal disease. Nevertheless, in vitro studies using the CRISPR-Cas9 as a gene editing tool are being conducted in order to treat corneal diseases and dystrophies. Human herpes viruses [373], TGFβ1-related corneal dystrophies [374], and the Schnyder corneal dystrophy [375] are great examples of this kind of in vitro studies.

There are many reasons why the cornea is a tissue of particular interest for targeted gene therapy. It is in part due to its accessibility, as this tissue consists of the outer layer of the eye. For example, eye drops can be very easily applied directly on the cornea in order to deliver all the CRISPR components. Furthermore, as the area of the cornea is quite restrained, only small amounts of these components are required. The cornea is also ideal for CRISPR-Cas9 use because of its immune privilege status and the absence of vascularity [376]. Indeed, the lack of blood vessels helps to reduce the immune response against the plasmids that are induced with the gene therapy [377]. The absence of vascularity is also of great interest because it nearly eliminates the possibility of the therapy reaching other organs.

Because the hTEC shares these features with the native cornea, it is also a great model to study gene therapy in vitro. As the hTEC needs to be maintained at the air–liquid interface for several days, the CRISPR-Cas9 components can be deposited directly on the humid upper surface of the hTEC and will not be diluted in the culture media. It is then easy to know the exact amount of components that are in contact with the tissue. The CRISPR-Cas9-targeted gene alteration can then be passed to the daughter cells of the superficial epithelial layers by simply raising the hTEC to the air–liquid interface. Another way of using the CRISPR-Cas9 technology with the hTEC would be to transfect the cells as monolayers prior to reconstructing the 3D model. However, it is important to make sure that the gene editing process does not affect cell proliferation and differentiation properties in order to produce a tissue-engineered cornea with a well-differentiated epithelium.

In the last decades, the progress made in cell culture and tissue engineering has resulted in the improvement of human corneal living substitutes. The success of these cultured tissues in some of the experimental and clinical applications presented in this review will pave the way for multiple avenues of fundamental and translational research. The improved living substitutes containing additional cell types (inflammatory and nerve cells) will help to unravel the complex molecular mechanisms and cellular pathways ongoing in the normal cornea and those leading to diseases. In addition, numerous applications will be concretized for the use of human corneal living substitutes as tools for pharmacotoxicologic studies and the development of new drugs. We hope that the clinical applications derived from the various living substitutes will improve the treatment of several forms of corneal blindness and favor a better quality of life for patients suffering from ocular diseases.

## 6. Patents

Sylvain L. Guérin, Lucie Germain, Karine Zaniolo, Camille Couture, and Pascale Desjardins (2015). Compositions favoring wound repair. WO2017075715A1, CA3003757A1, EP3370717A4, US10537547. Assignee: Université Laval.

Élodie Boisselier, Mathieu Ouellette, Vincent Pernet, and Mahmoud Omar (2016). Ultra-stable gold nanoparticles for drug delivery applications and synthesis thereof. WO2018102921A1, CA3043775A1, US20190374478A1. Assignee: Université Laval.

## Data Availability

No new data were created or analyzed in this study. Data sharing is not applicable to this review article.
